# The effect of losartan therapy on ventricular function in Marfan patients with haploinsufficient or dominant negative *FBN1* mutations

**DOI:** 10.1007/s12471-016-0905-8

**Published:** 2016-10-04

**Authors:** A. W. den Hartog, R. Franken, M. P. van den Berg, A. H. Zwinderman, J. Timmermans, A. J. Scholte, V. de Waard, A. M. Spijkerboer, G. Pals, B. J. M. Mulder, M. Groenink

**Affiliations:** 1 Department of Cardiology, Academic Medical Center Amsterdam, Rm. F3-147, Meibergdreef 9, 1105 AZ Amsterdam, The Netherlands; 2 Institute of the Netherlands, Interuniversity Cardiology, Utrecht, The Netherlands; 3 Department of Cardiology, University Medical Center Groningen, Groningen, The Netherlands; 4 Department of Clinical Epidemiology and Biostatistics, Academic Medical Center Amsterdam, Amsterdam, The Netherlands; 5 Department of Cardiology, Radboud University Medical Center Nijmegen, Nijmegen, The Netherlands; 6 Department of Cardiology, Leiden University Medical Center, Leiden, The Netherlands; 7 Department of Medical Biochemistry, Academic Medical Center Amsterdam, Amsterdam, The Netherlands; 8 Department of Radiology, Academic Medical Center Amsterdam, Amsterdam, The Netherlands; 9 Department of Clinical Genetics, VU University Medical Center, Amsterdam, The Netherlands

**Keywords:** Marfan syndrome, Ventricular function, Ventricular volumes, Losartan, Cardiac magnetic resonance imaging, *FBN1* mutation classes

## Abstract

**Background:**

Mild biventricular dysfunction is often present in patients with Marfan syndrome. Losartan has been shown to reduce aortic dilatation in patients with Marfan syndrome. This study assesses the effect of losartan on ventricular volume and function in genetically classified subgroups of asymptomatic Marfan patients without significant valvular regurgitation.

**Methods:**

In this predefined substudy of the COMPARE study, Marfan patients were classified based on the effect of their *FBN1* mutation on fibrillin-1 protein, categorised as haploinsufficient or dominant negative. Patients were randomised to a daily dose of losartan 100 mg or no additional treatment. Ventricular volumes and function were measured by magnetic resonance imaging at baseline and after 3 years of follow-up.

**Results:**

Changes in biventricular dimensions were assessed in 163 Marfan patients (48 % female; mean age 38 ± 13 years). In patients with a haploinsufficient *FBN1 *mutation (*n* = 43), losartan therapy (*n* = 19) increased both biventricular end diastolic volume (EDV) and stroke volume (SV) when compared with no additional losartan (*n* = 24): left ventricular EDV: 9 ± 26 ml vs. −8 ± 24 ml, *p* = 0.035 and right ventricular EDV 12 ± 23 ml vs. −18 ± 24 ml; *p* < 0.001 and for left ventricle SV: 6 ± 16 ml vs. −8 ± 17 ml; *p* = 0.009 and right ventricle SV: 8 ± 16 ml vs. −7 ± 19 ml; *p* = 0.009, respectively. No effect was observed in patients with a dominant negative *FBN1* mutation (*n* = 92), or without an *FBN1* mutation (*n* = 28).

**Conclusion:**

Losartan therapy in haploinsufficient Marfan patients increases biventricular end diastolic volume and stroke volume, furthermore, losartan also appears to ameliorate biventricular filling properties.

## Introduction

Marfan syndrome (MFS) is caused by mutations in the *FBN1* gene, leading to deficiency (haploinsufficient {HI} mutations) or malformation (dominant-negative {DN} mutations) of the fibrillin-1 protein [1], ultimately leading to aortic dilatation and dissection [2–4]. Treatment with losartan, an angiotensin-II receptor-1 blocker (ARB), has been shown to have a beneficial effect on aortic pathology in MFS murine models. Human MFS studies on aortic dilatation rate have demonstrated conflicting results [5–7], however, losartan may have a beneficial effect in patients with an HI mutation.

Although a mild biventricular dysfunction is often present in MFS patients without significant valvular pathology [8–12], the effect of losartan on cardiac dimensions and function in MFS patients is still unknown. Here, we assess the effect of losartan on left and right ventricular dimensions and function in both HI and DN asymptomatic MFS patients without significant valvular regurgitation by cardiac magnetic resonance imaging (CMR).

## Methods

### Patients

All study subjects were participants in the multicentre, randomised COMPARE trial [5, 13]. In short, the COMPARE trial was designed to investigate the effect of losartan on the aortic dilatation rate in adult MFS patients. Patients (≥18 years) fulfilling the Ghent criteria of 1996 were included [14]. Exclusion criteria were: (A) treatment with angiotensin-converting-enzyme inhibitor (ACEi) or ARB before study entry, (B) an aortic root diameter >50 mm, (C) a history of aortic dissection, and (D) ≥1 vascular prosthesis. For the current predefined study, (E) only patients fulfilling the revised Ghent criteria of 2010 [15] were included, and (F) patients with moderate to severe valvular regurgitation were excluded [16]. All previously prescribed medication, including β‑blockers and calcium channel blockers, was continued during the study. Written informed consent was obtained from all participants. The study protocol conforms to the ethical guidelines of the 1975 Declaration of Helsinki and was approved by the institution’s human research committee. All patients gave their informed consent.

### Mutation classification

To detect mutations, sanger sequencing of the 65 coding *FBN1* exons in genomic DNA was used. Mutations were classified into two groups according to the effect of the mutation on the fibrillin-1 protein [17]. Patients with a DN effect were defined as having mutations leading to production of malfunctional fibrillin-1 protein. DN mutations included: (1) missense mutations leading to stable mutant fibrillin-1 protein; (2) mutations leading to exon skipping or deletion resulting in in-frame events [18]; and (3) premature termination codon mutations leading to a shorter but stable fibrillin-1 protein (>10 exons).

An HI effect of the *FBN1* mutation was defined as a mutation leading to a reduced amount of normal fibrillin-1 protein [19–21] as predicted by the Alamut® software (Interactive Biosoftware, Rouen, France) [21]. HI mutations included: (1) deletion of the whole *FBN1 *gene; (2) deletion of at least the first or last exon; (3) premature termination codon mutations leading to nonsense mediated decay or to a very short truncated protein (translation <10 exons of the *FBN1 *gene) [22]; and (4) missense mutations leading to degradation of the protein [23].

### Medication

Participating patients started on losartan treatment after baseline examinations. Patients were randomly assigned to a 1:1 ratio to receive losartan daily (losartan group), or to no additional treatment (control group). Target dosage was 100 mg losartan daily.

### Magnetic resonance imaging

At baseline and after 3 years of follow-up, CMR imaging of the right and left ventricle was performed. All CMR scans were performed at two centres (Academic Medical Center Amsterdam and Leiden University Medical Center). To obtain ventricular volumes, magnetic resonance imaging was performed with either an Avanto (Siemens, Erlangen, Germany), or a Philips (Intera, release 11 and 12; Philips Medical Systems, Best, the Netherlands) 1.5 T MRI scanner using a phased array cardiac receiver coil. ECG-gated cine images were acquired during breath-hold using a segmented, steady-state, free-precession sequence. Short-axis views were obtained every 10 mm, starting from the base up to the apex and covering the entirety of both ventricles. Additionally, flow (m/s) through the aortic root and ascending aorta was assessed by phase contrast velocity mapping to rule out significant valvular regurgitation, unnoticed at prior echocardiography.

### Image analysis

The primary endpoint of this study was change in left and right ventricular volumes and systolic function after 3 years of follow-up measured by CMR. All measurements were performed using MASS® and FLOW® image analysis software (Medis, Leiden, the Netherlands) by a single observer (AWdH) under the supervision of an experienced cardiologist (MG) and radiologist (AMS) with no prior knowledge of patients’ medical therapy.

Endocardial contours of the left and right ventricle were manually traced in end systole and end diastole on all short-axis images. End diastole was defined as the phase with the largest ventricular area and end systole as the phase with the smallest ventricular area. Left ventricular (LV) and right ventricular (RV) end diastolic volume (EDV), end systolic volume (ESV) and stroke volume (SV = EDV-ESV) were determined. Left and right ventricular ejection fraction (EF) was calculated (EF = SV/EDV). Flow through the aorta was calculated using manually drawn areas on the modulus images and the velocity values of the corresponding velocity encoded images by using flow image analysis software (Flow®, Medis, Leiden, the Netherlands). Severity of valvular regurgitation was assessed quantitatively by CMR in accordance with ACC/AHH guidelines for the management of patients with valvular heart disease [16]. Moderate to severe valvular regurgitation was defined as regurgitant volume of ≥30 ml per beat or a regurgitant fraction of ≥30 % [16]. a dilated ventricle was assumed to exceed an EDV of 97 ml/m^2^ [24].

### Statistical analysis

Data are presented as mean value ± standard deviation and categorical variables as numbers and percentages. Between-group comparisons of quantitative and categorical variables were made by Students’ t‑test and Fisher’s exact tests, respectively. Associations between characteristics were explored with regression analysis. The effect of losartan on change in ventricular volumes and function was evaluated by the two-tailed Student’s t‑tests. All analyses were per protocol analyses in order to evaluate the change in ventricular volumes between the two groups of MFS patients who continued their losartan treatment throughout the entire study, and those in whom losartan treatment was not started during the study. a two-tailed *P* < 0.05 was considered statistically significant. For statistical analyses, PASW 19.0 (SPSS Inc., Chicago, IL) for Windows (Microsoft, Redmond, WA) was used.

## Results

### Patients

**Fig. 1:**
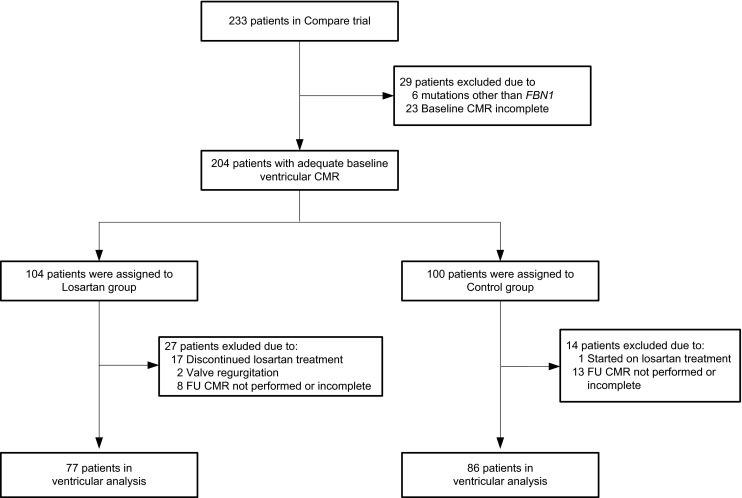
Randomisation and follow-up for ventricular function analysis. Abbreviations: *CMR* denotes cardiac magnetic resonance, *FU* denotes follow-up

Patient recruitment and characteristics have been described in detail previously [5]. (17)In the COMPARE trial, a total of 233 MFS patients were included between January 2008 and December 2009 [5]. Change in cardiac function was assessed in 163 patients (48 % female, mean age 38 ± 13 years, Fig. 1). a pathogenic *FBN1 *mutation was found in 135 patients (83 %). In patients with a known *FBN1 *mutation, 92 patients (68 %) had a DN *FBN1* mutation and 43 patients (32 %) had an HI *FBN1* mutation. No *FBN1* mutation could be found in 28 patients. a total of 77 MFS patients were randomised to losartan treatment, while 86 MFS patients received no additional treatment. The remaining cardiovascular medicinal treatment did not change during follow-up.

Baseline characteristics between the treatment groups were comparable with the exception of body surface area (BSA) and heart rate in HI patients (Table 1).

**Table 1:** Baseline characteristics of 163 patients with Marfan syndrome

Characteristics	Losartan			No losartan		
	No *FBN1*	DN	HI	No *FBN1*	DN	HI
Number of patients	16	42	19	12	50	24
Age (years)	39 ± 13	36 ± 12	33 ± 10	44 ± 12	39 ± 14	37 ± 13
Male (%)	9 (56)	23 (55)	10 (53)	6 (50)	24 (48)	13 (54)
BSA (m^2^)	2.0 ± 0.2	2.0 ± 0.2	2.0 ± 0.2*	1.9 ± 0.4	2.0 ± 0.2	2.1 ± 0.2*
MAP (mm Hg)	93 ± 13	91 ± 11	91 ± 11	87 ± 13	90 ± 9	90 ± 8
Heartrate (bpm)	67 ± 11	68 ± 13	67 ± 14*	68 ± 15	66 ± 11	61 ± 6*
Aortic root diameter (mm)	45 ± 6	44 ± 5	44 ± 6	42 ± 3	45 ± 5	43 ± 5
Aortic root replacement (%)	4 (25)	6 (14)	5 (26)	7 (58)	16 (32)	5 (21)
Mitral valve surgery (%)	1 (6)	0	0	0	4 (8)	0
*Valve dysfunction*						
≥ moderate AR (%)	0	0	1 (3)	0	0	0
≥ moderate MR (%)	0	0	1 (3)	0	0	0
≥ moderate TR (%)	0	0	0	0	0	0
≥ moderate PR (%)	0	0	0	0	0	0
*Medication*						
β-blocker use (%)	13 (81)	31 (74)	14 (74)	9 (75)	36 (72)	14 (58)
*Left Ventricular*						
ESV/BSA (ml/m^2^)	39 ± 9	42 ± 11	42 ± 10	44 ± 13	44 ± 14	39 ± 9
EDV/BSA (ml/m^2^)	81 ± 14	86 ± 18	87 ± 17	89 ± 19	88 ± 18	86 ± 15
SV/BSA (ml/m^2^)	43 ± 8	44 ± 10	45 ± 9	45 ± 8	44 ± 7	46 ± 8
EF (%)	53 ± 7	51 ± 6	52 ± 4	52 ± 6	50 ± 7	54 ± 5
*Right Ventricular*						
ESV/BSA (ml/m^2^)	45 ± 12	40 ± 12	41 ± 10	43 ± 11	42 ± 16	40 ± 10
EDV/BSA (ml/m^2^)	86 ± 19	82 ± 18	83 ± 14	86 ± 18	84 ± 19	85 ± 13
SV/BSA (ml/m^2^)	41 ± 9	42 ± 8	42 ± 7	43 ± 9	42 ± 7	45 ± 7
EF (%)	48 ± 5	52 ± 7	51 ± 5	50 ± 6	51 ± 8	53 ± 7

*BSA* Body Surface Area, *MAP* Mean Arterial Pressure, *AR* Aortic Regurgitation, *MR* Mitral Regurgitation, *TR* Tricuspid Regurgitation, *PR* Pulmonary Regurgitation, *ESV* End Systolic Volume, *EDV* End Diastolic Volume, *SV* Stroke Volume, *EF* Ejection Fraction, *CI* Cardiac Index, *DN* Dominant Negative FBN1 mutation, *HI* Haploinsufficient FBN1 mutation

Plus-minus values are means ± SD

^*^ *p* < 0.005 for BSA and Heartrate in HI patients between treatment groups

Left ventricular EF in DN patients was lower than the EF in HI patients, 51 ± 7 % vs. 53 ± 4 %, *p* = 0.013. The remaining ventricular characteristics were comparable between the groups, including the frequency of a dilated ventricle 26 % vs. 23 %, *p* = 0.724, respectively.

### Losartan and ventricular function in mutational subgroups

**Fig. 2:**
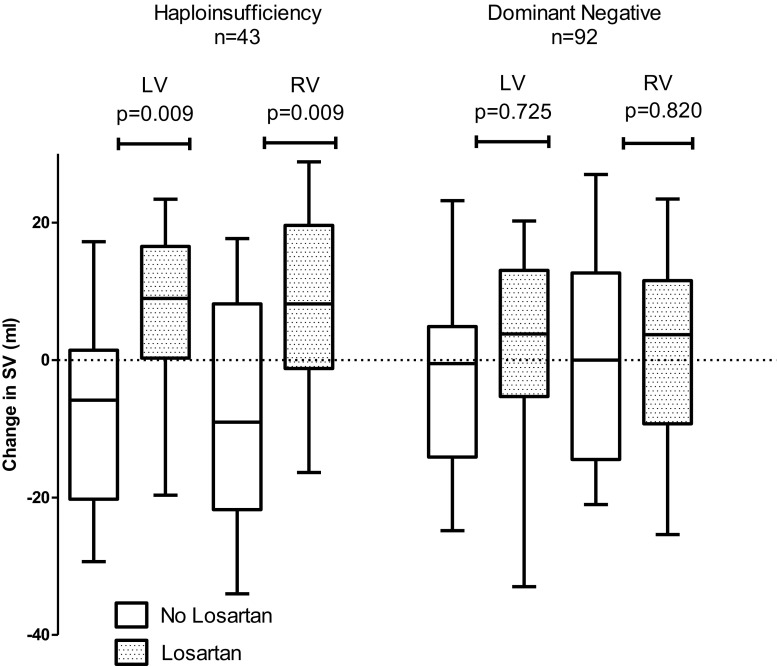
Box plot showing change in left and right ventricular stroke volume between control and losartan group in Marfan patients with a *FBN1* mutation leading to haploinsufficiency (*n* = 43) or to a dominant-negative effect (*n* = 92). Abbreviations: *SV* denotes stroke volume, *LV* denotes left ventricle, *RV* denotes right ventricle. *Boxes* indicate median with interquartile range. *Error bars* indicate upper 90 percentile and lower 10 percentile limits

After a mean of 3.1 ± 0.4 years of follow-up, the biventricular EDV increased in HI patients on losartan therapy (*n* = 19) compared with HI controls (*n* = 24): LV-EDV: 9 ± 26 ml vs. −8 ± 24 ml; *p* = 0.035 and RV-EDV 12 ± 23 ml vs. −18 ± 24 ml; *p* < 0.001, respectively. Similar results were seen in RV-ESV, however, not in LV-ESV (Table 2). In HI patients on losartan therapy, the increase in biventricular EDV was accompanied by an increase in SV, for LV-SV: 6 ± 16 ml vs. −8 ± 17 ml; *p* = 0.009 and RV-SV: 8 ± 16 ml vs. −7 ± 19 ml; *p* = 0.009, respectively (Table 2, Fig. 2). No effect of losartan was observed in patients with a DN *FBN1* mutation or without an *FBN1* mutation. No effect of losartan on mean arterial blood pressure (MAP) was observed in HI patients.

**Table 2:** The effect of losartan on left and right ventricle volume and function in Marfan patients with a FBN1 mutation leading to haploinsufficiency or a dominant-negative effect during 3 years of follow-up

	Haploinsufficiency			Dominant Negative		
	Losartan	No losartan	*p*-value	Losartan	No losartan	*p*-value
Number of patients	19	24	–	42	50	–
∆ MAP (mm Hg)	−3 ± 8	−4 ± 9	0.684	−8 ± 10	−2 ± 8	0.003
∆ HR (bpm)	3 ± 16	4 ± 23	0.950	0 ± 21	3 ± 15	0.442
∆ *Left Ventricular*						
ESV (ml)	3 ± 16	0 ± 15	0.537	4 ± 22	2 ± 22	0.696
EDV (ml)	9 ± 26	−8 ± 24	0.035	3 ± 33	0 ± 29	0.637
SV (ml)	6 ± 16	−8 ± 17	0.009	−1 ± 19	−2 ± 16	0.725
EF (%)	1 ± 5	−2 ± 6	0.157	−1 ± 6	−1 ± 7	0.770
∆ *Right Ventricular*						
ESV (ml)	4 ± 20	−12 ± 13	0.004	−1 ± 22	−8 ± 27	0.116
EDV (ml)	12 ± 23	−18 ± 24	<0.001	0 ± 31	−8 ± 27	0.223
SV (ml)	8 ± 16	−7 ± 19	0.009	1 ± 17	0 ± 17	0.820
EF (%)	1 ± 7	1 ± 7	0.889	1 ± 7	2 ± 7	0.418

*MAP* Mean Arterial Pressure, *HR* Heartrate, *ESV* End Systolic Volume, *EDV* End Diastolic Volume, *SV* Stroke Volume, *EF* Ejection Fraction

Plus-minus values are means ± SD

Changes in left and right ventricular volumes and function in the entire cohort were comparable between the losartan group and the control group. Furthermore, analysis of losartan only therapy or a combination of losartan and β‑blocker therapy on right or left ventricular volumes, heart rate or EF in this subgroup rendered similar results.

### Blood pressure and ventricular function

In the entire cohort, after 3 year’s treatment losartan significantly reduced MAP by 7 ± 10 mm Hg when compared with baseline (*p* < 0.001), and differed significantly from change in MAP compared with the control group. At baseline, no significant correlation was found between MAP and ESV, EDV or SV (*r = *0.064, *p* = 0.426; *r =* −0.014, *p* = 0.860; *r =* −0.108, *p* = 0.176; respectively). In losartan treated patients, there was also no significant correlation between change in MAP and change in ESV, EDV or SV (*r =* −0.117, *p* = 0.326; *r =* −0.060, *p* = 0.615; *r = *0.023, *p* = 0.847; respectively).

## Discussion

To our knowledge, this is the first large follow-up study to assess the effect of losartan on biventricular function in both HI and DN asymptomatic MFS patients using CMR. In this study, LV-EF was lower in MFS patients with a DN *FBN1* mutation than in those with an HI *FBN1* mutation. Losartan therapy increased EDV and SV, independent of change in blood pressure only in MFS patients with an HI *FBN1* mutation. Furthermore, no beneficial effect of losartan on cardiac systolic function in the study group as a whole could be demonstrated.

MFS is caused by mutations in the *FBN1* gene, leading to HI or DN mutations of the fibrillin-1 protein [1]. Both mutations indirectly lead to increased levels of TGF-β, which is correlated to aortic disease in MFS [17]. However, TGF-β most likely also plays a role in myocardial fibrosis [25, 26]. Furthermore, blocking of TGF-β in both rat studies involving pressure-overloaded hearts and mice studies involving hypertrophic hearts, prevented myocardial fibrosis and diastolic dysfunction [27–29].

Losartan, an angiotensin II receptor type 1 inhibitor, is known to reduce both TGF-β signalling and blood pressure. However, we demonstrated that (change of) mean arterial blood pressure did not correlate with (change in) ventricular dimensions. Therefore, the effect of losartan on ventricular dimensions is not merely blood pressure-related. a possible explanation for the significant difference in baseline ventricular function and response to losartan between HI and DN might therefore be due to a difference in TGF-β levels. This is in line with an earlier study by Franken et al., who demonstrated that losartan therapy significantly reduced the aortic root dilatation rate in the HI, whereas only a modest, insignificant reduction was found in DN MFS patients [17]. Furthermore, HI patients have a reduced but functionally normal fibrillin-1 protein, while the DN patient group is more heterogeneous since they display a broad spectrum of dysfunctional fibrillin-1 proteins, possibly explaining the variable response to losartan in the DN group. In addition, reduced ventricular function in DN patients might also be the result of a disorganised ECM due to the dysfunctional fibrillin-1 proteins [30, 31].

In conclusion, systolic ventricular function in MFS patients with a DN *FBN1* mutation was slightly, but significantly reduced compared with patients with an HI *FBN1* mutation. In MFS patients with an *FBN1* mutation leading to HI, losartan increased both EDV and SV, independent of change in blood pressure, suggesting a beneficial effect on left and right ventricular filling properties. No beneficial effect of losartan on ventricular function in the entire MFS cohort could be demonstrated.

## Limitations

Our patients were still relatively young and mainly asymptomatic. Furthermore, the clinical implications of our findings are limited. However, the occurrence of ventricular dysfunction will most likely increase due prolonged longevity in MFS as a result of prophylactic aortic root replacement and improved medicinal therapy. Other limitations are the limited sample size, lack of TGF-β levels and relatively small subgroups. Although losartan appeared to increase EDV in HI patients, thereby suggesting a beneficial effect on left and right ventricular filling properties, absolute diastolic filling properties were not assessed by CMR.
